# Metabolomic analysis of uremic pruritus in patients on hemodialysis

**DOI:** 10.1371/journal.pone.0246765

**Published:** 2021-02-12

**Authors:** Christian G. Bolanos, Nhat M. Pham, Robert D. Mair, Timothy W. Meyer, Tammy L. Sirich

**Affiliations:** 1 The Department of Medicine, Stanford University, Palo Alto, California, United States of America; 2 The Department of Medicine, VA Palo Alto Healthcare System, Palo Alto, California, United States of America; 3 The Department of Medicine, Santa Clara Valley Medical Center, San Jose, California, United States of America; CIC bioGUNE, SPAIN

## Abstract

Pruritus is a common debilitating symptom experienced by hemodialysis patients. Treatment is difficult because the cause of uremic pruritus is not known. This study addressed the hypothesis that pruritus is caused by solutes that accumulate in the plasma when the kidneys fail. We sought to identify solutes responsible for uremic pruritus using metabolomic analysis to compare the plasma of hemodialysis patients with severe pruritus versus mild/no pruritus. Pruritus severity in hemodialysis patients was assessed using a 100-mm visual analogue scale (VAS), with severe pruritus defined as >70 mm and mild/no pruritus defined as <10 mm. Twelve patients with severe pruritus (Itch) and 24 patients with mild/no pruritus (No Itch) were included. Pre-treatment plasma and plasma ultrafiltrate were analyzed using an established metabolomic platform (Metabolon, Inc.). To identify solutes associated with pruritus, we compared the average peak area of each solute in the Itch patients to that of the No Itch patients using the false discovery rate (q value) and principal component analysis. Dialysis vintage, Kt/V_urea_, and serum levels of calcium, phosphorus, PTH, albumin, ferritin, and hemoglobin were similar in the Itch and No Itch patients. Metabolomic analysis identified 1,548 solutes of which 609 were classified as uremic. No difference in the plasma or plasma ultrafiltrate levels of any solute or group of solutes was found between the Itch and No Itch patients. Metabolomic analysis of hemodialysis patients did not reveal any solutes associated with pruritus. A limitation of metabolomic analysis is that the solute of interest may not be included in the metabolomic platform’s chemical library. A role for uremic solutes in pruritus remains to be established.

## Introduction

Pruritus is common in hemodialysis patients and is associated with poor quality of life and increased mortality [1, 2]. The mechanism is incompletely known and treatment options are limited. A DOPPS study identified several factors associated with pruritus including longer dialysis vintage, male gender, spKt/V_urea_ lower than 1.5, higher serum levels of calcium and phosphorus, and lower serum levels of albumin, ferritin, and hemoglobin [1]. Other studies have confirmed some of these associations while obtaining divergent results for the associations with pruritus of lower spKt/V_urea_ values and higher serum levels of calcium and phosphorus [1, 3].

An important potential contributor to pruritus is the accumulation of uremic solutes. Perhaps the strongest evidence implicating these solutes is that pruritus resolves following kidney transplantation [4]. If we could identify solutes that cause pruritus, therapies (either dialytic or non-dialytic) could be tailored to reduce their plasma levels. This study employed untargeted mass spectrometry (metabolomics) to assess levels of numerous solutes in individual plasma samples [5]. Solute levels in plasma from hemodialysis patients with severe pruritus were compared with those in patients with minimal or no pruritus.

## Materials and methods

### Patient recruitment

Hemodialysis patients in Northern California were recruited from August 2018 to July 2019. Hemodialysis patients were included if they had been maintained on hemodialysis for at least two months and were greater than 18 years old. Patients were excluded if they had residual kidney function (measured residual urea clearance >2.5 ml/min or report of urine output >2 cups per day), were hospitalized or on antibiotics during the prior two months, had diagnosed skin disease, or had liver cirrhosis. Subjects with no known kidney disease were included as controls. Informed consent was obtained from all participants. The study was approved by the Stanford Institutional Review Board and was conducted in accordance with the Declaration of Helsinki.

Hemodialysis patients were visited at a midweek session for assessment of pruritus and symptom burden and for blood sample collection. Pruritus was assessed using the 100-mm visual analogue scale (VAS) [6]. Symptom burden was assessed using the Dialysis Symptom Index (DSI) [7]. A pre-treatment blood sample was collected on the same day as the VAS and DSI assessments. Patients were then divided into two groups for metabolomic analysis based on their VAS score–severe pruritus defined as VAS > 70 mm (Itch) and mild/no pruritus defined as VAS < 10 mm (No Itch). The wide separation of groups by pruritus severity was chosen to increase the likelihood of identifying solutes associated with pruritus. The patients with VAS scores outside of the Itch and No Itch criteria were not analyzed further. Clinical laboratory data was obtained from dialysis unit records.

**Fig 1:**
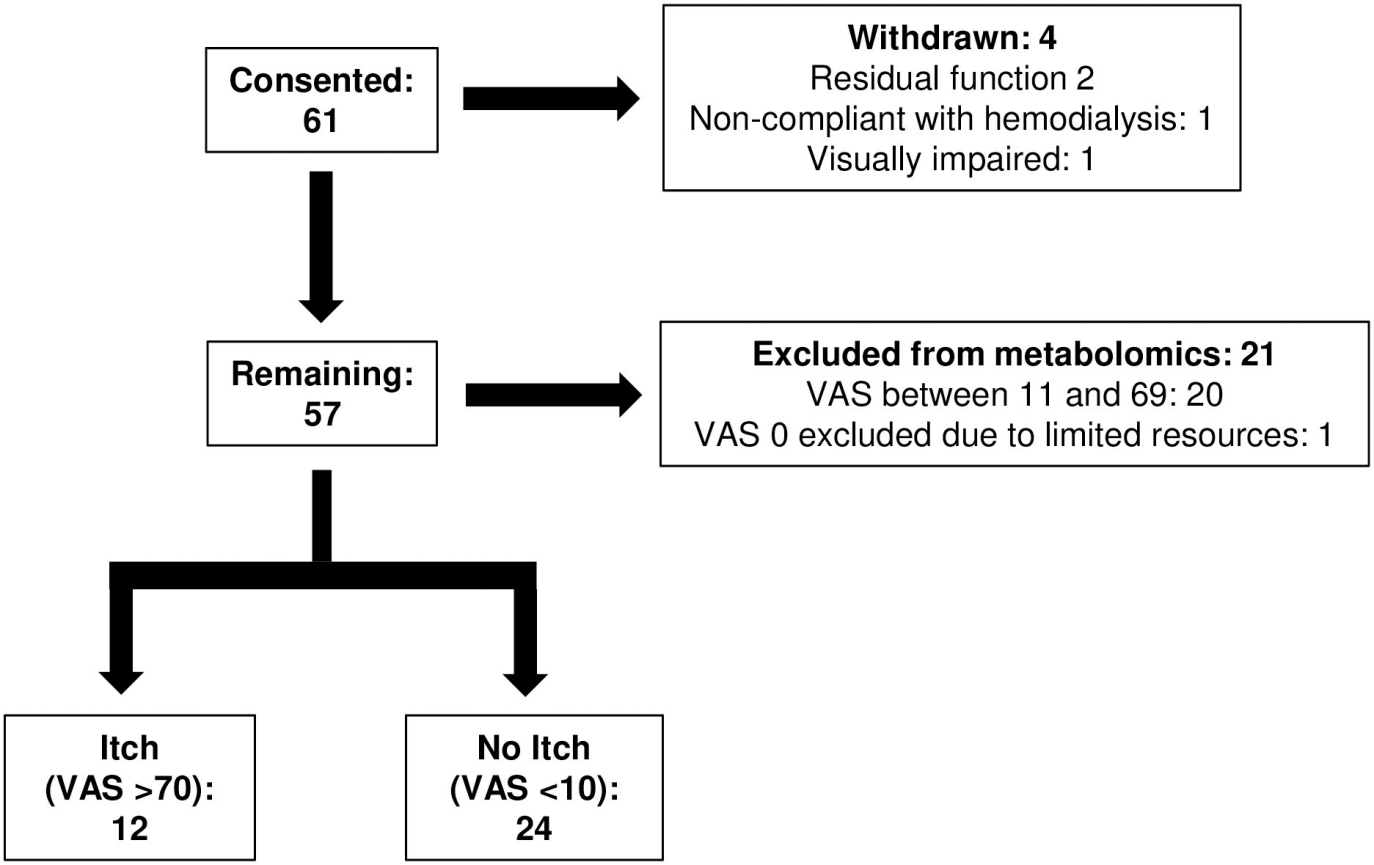
Patient enrollment flow chart. Of 61 patients, 12 patients with severe pruritus (Itch, VAS >70 mm) and 24 patients with mild/no pruritus (No Itch, VAS <10 mm) were included for metabolomic analysis. VAS, visual analogue scale.

Patient enrollment is illustrated in Fig 1. 61 patients were consented of whom four were later withdrawn–two patients were found to have residual kidney function, one missed hemodialysis treatments, and one was visually impaired and could not complete the VAS. Histogram of the VAS scores for the remaining 57 patients is provided in S1 Fig. To obtain the maximal separation in VAS scores with the resources available for metabolomic analysis, we included 12 patients in the Itch group and 24 patients in the No Itch group. The remaining 21 patients were excluded from metabolomic analysis– 20 patients had intermediate VAS scores (average VAS 35±14, range 13 to 66) and one patient with a VAS score of 0 was not included in the metabolomic analysis due to limited resources. Blood samples obtained from 16 control subjects without known kidney disease were analyzed to enable us to identify uremic solutes as defined by their higher levels in the hemodialysis patients.

### Sample processing

Blood samples were collected using K2EDTA tubes and plasma was obtained by centrifugation at 3,000 rpm for 10 minutes. Plasma ultrafiltrate was obtained using Nanosep 30K Omega separators (Pall, Ann Arbor, MI). Plasma and ultrafiltrate samples were stored in a -80 °C freezer and sent to Metabolon, Inc. for metabolomic analysis using a liquid chromatography mass spectrometry platform as previously described [8, 9].

### Identification of uremic solutes

A total of 1,548 solutes were identified in the plasma and ultrafiltrate samples of the hemodialysis patients. Of these solutes, 823 were detected in both plasma and ultrafiltrate, 618 were detected in only plasma, and 107 were detected in only ultrafiltrate. We identified uremic solutes by comparing the average peak area of each solute in the 36 hemodialysis patients and the 16 control subjects (hemodialysis to control ratio). The hemodialysis to control ratios were calculated separately for the plasma and ultrafiltrate samples. When no peak area was reported by the mass spectrometer in an individual sample, we imputed a value half that of the lowest peak area detected in any sample of the same fluid type among the entire group of 36 hemodialysis patients and 16 control subjects. Solute peak areas of the hemodialysis patients and control subjects were log-transformed and then compared using the unpaired t-test. To correct for multiple comparisons, the false discovery rate (q value) was calculated [10]. 609 solutes were identified as uremic based on the following criteria:

- Hemodialysis to Control ratio >4 in plasma and/or ultrafiltrate
- q value < 0.05

### Metabolomic analysis of Itch versus No Itch hemodialysis patients

To determine if any of the 609 uremic solutes were associated with pruritus, we compared the average peak area of each solute in the plasma and in the ultrafiltrate of the 12 Itch hemodialysis patients to that of the 24 No Itch hemodialysis patients. Solute peak areas of the Itch and No Itch hemodialysis patients were log-transformed and then compared using the unpaired t-test, followed by the false discovery rate. Principal component analysis (PCA) was then employed to determine whether a combination of uremic solutes was associated with pruritus. These analyses were performed separately for the plasma and ultrafiltrate samples. We also compared all 1,548 solutes in the Itch and No Itch patients using the same methods.

### Statistical analysis

Characteristics and laboratory values of the Itch and No Itch patients were compared using the Mann Whitney U test and the chi-squared test (for gender, ethnicity, race, and co-morbidities) using SPSS software v24. False discovery rates (q values) were calculated using software from http://qvalue.princeton.edu [10]. PCA was performed using MetaboAnalyst 4.0 [11].

## Results

Characteristics of the 12 Itch and 24 No Itch hemodialysis patients are summarized in Table 1. As per the study design, the Itch patients had significantly higher VAS scores than the No Itch patients (87±9 vs. 1.5±2.0). The DSI scores were likewise significantly higher in the Itch than the No Itch patients, indicating greater overall symptom burden in the patients with severe pruritus (46±16 vs. 22±15, p <0.001). The Itch patients were on average older than the No Itch patients (69±9 vs. 56±12 years, p 0.002). There was a trend towards lower serum creatinine level and lower nPCR in the Itch patients, but the difference did not meet statistical significance. These trends could be related to the older age of the Itch patients, as creatinine and nPCR tended to fall with increasing age (S2 Fig). Dialysis vintage, spKt/V_urea_ values, gender, ethnicity, race, co-morbidities, weight, body mass index, and body surface area were similar between the two groups. There was no difference in the seasonal timing of study participation between the two groups, as summarized in S1 Table. Other clinical laboratory data from the patients’ most recent monthly tests prior to the study were likewise similar between the two groups, as summarized in Table 2.

**Table 1:** Characteristics of Itch and No Itch HD patients.

		Itch	No Itch	p value
		(n = 12)	(n = 24)	
VAS (mm)		87 ± 9	1.5 ± 2.0	<0.001
DSI		46 ± 16	22 ± 15	<0.001
Age (years)		69 ± 9	56 ± 12	0.002
Creatinine (mg/dl)		9.4 ± 2.4	11.2 ± 2.9	0.067
nPCR (g/kg/day)		1.0 ± 0.2	1.2 ± 0.3	0.062
Dialysis vintage (years)		7 ± 3	7 ± 5	0.78
spKt/V_urea_		1.56 ± 0.13	1.61 ± 0.21	0.52
Men / Women (#)		7 / 5	20 / 4	0.10
Hispanic (#, %)		6 (50)	15 (63)	0.47
Race (#, %)	White	8 (67)	18 (75)	0.60
	Black	1 (8)	2 (8)	1.0
	Other	3 (25)	4 (17)	0.55
Co-Morbidities (#, %)	Diabetes	10 (83)	13 (54)	0.086
	Hypertension	11 (92)	21 (88)	0.71
	Cardiovascular disease	3 (25)	5 (21)	0.78
	Congestive heart failure	5 (42)	7 (29)	0.45
	Cerebral vascular disease	3 (25)	4 (17)	0.55
Weight (kg)		78 ± 19	75 ± 19	0.48
BMI (kg/m^2^)		30 ± 7	25 ± 5	0.053
BSA (m^2^)		1.8 ± 0.2	1.9 ± 0.3	0.73

Results are mean ± standard deviation. VAS, visual analogue scale; DSI, Dialysis Symptom Index; BMI, body mass index; BSA, body surface area. BSA was calculated using the Dubois equation. Characteristics between the two groups were compared using the Mann Whitney U test and the chi-squared test (for gender, ethnicity, race, and co-morbidities). One patient in the No Itch group declined to complete the DSI.

**Table 2:** Laboratory data of Itch and No Itch HD patients.

	Itch (n = 12)	No Itch (n = 24)	p value
Ca (mg/dl)	9.1 ± 0.9	9.0 ± 0.9	0.54
Phosphorus (mg/dl)	5.1 ± 1.7	6.0 ± 2.2	0.14
Ca x Phos Product	46 ± 16	51 ± 21	0.28
PTH (pg/ml)	364 ± 236	363 ± 227	1.0
Albumin (g/dl)	4.0 ± 0.4	4.0 ± 0.4	0.86
Ferritin (ng/dl)	733 ± 314	625 ± 279	0.44
Serum Sodium (mEq/L)	139 ± 3	137 ± 3	0.24
Serum Potassium (mEq/L)	4.9 ± 0.7	5.1 ± 0.7	0.40
Hemoglobin (g/dl)	11.1 ± 1.0	11.3 ± 1.2	0.52

Results are mean ± standard deviation. All patients received thrice weekly hemodialysis except for one patient in the Itch group who was on four times weekly hemodialysis. Laboratory data represents the patients’ most recent monthly clinical tests prior to the study. The time period between the laboratry data and the study was 15±11 days for the Itch patients and 14±10 days for the No Itch patients. Laboratory data between the two groups was compared using the Mann Whitney U test.

**Fig 2:**
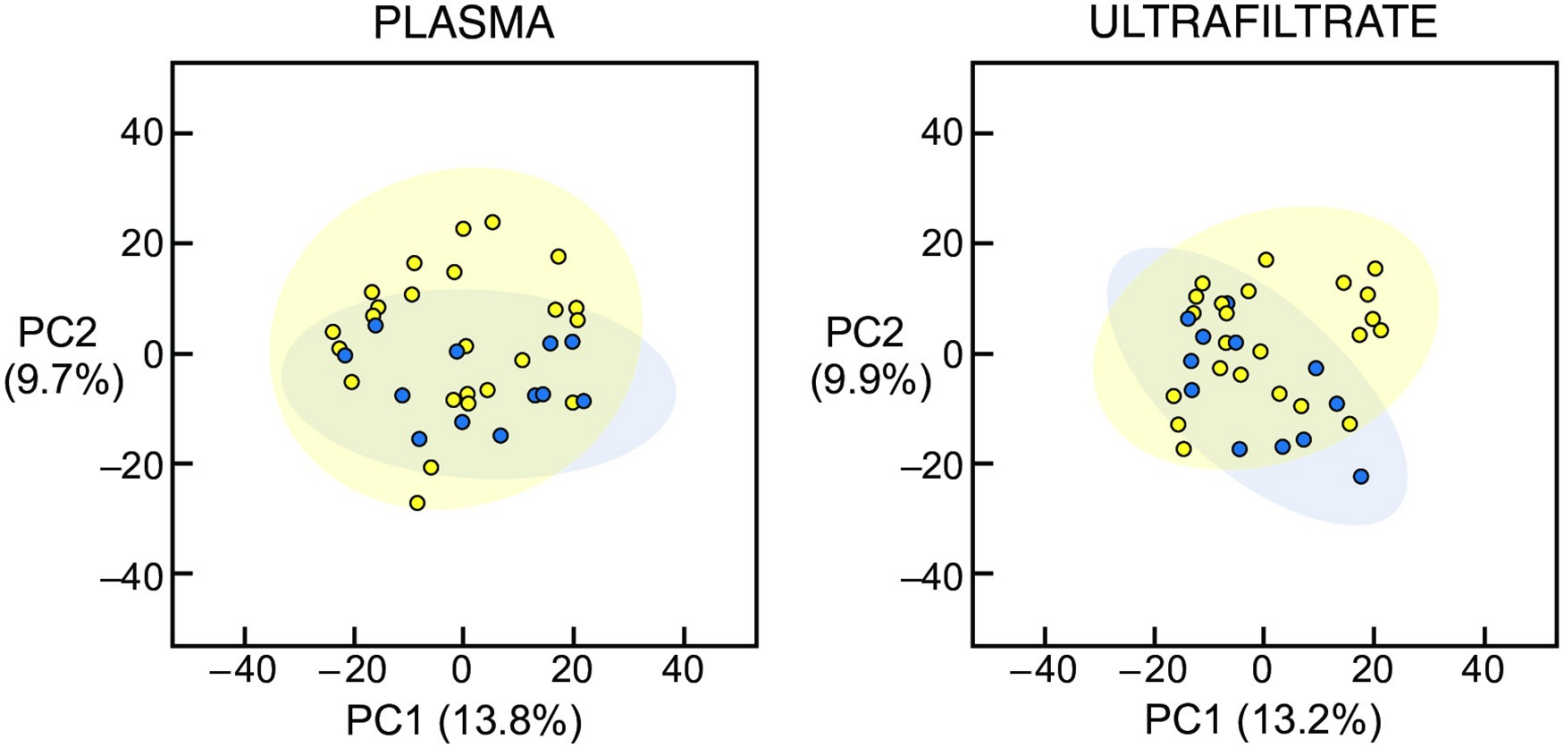
Principal component analysis of uremic solutes in plasma and ultrafiltrate of hemodialysis patients. Principal component analysis score plots of the uremic solute profile in the plasma (left panel) and ultrafiltrate (right panel) are illustrated. There was no difference in the uremic solute profile between the 12 Itch patients (blue circles) and the 24 No Itch patients (yellow circles). PC1, principal component 1; PC2, principal component 2.

Metabolomic analysis of the plasma and ultrafiltrate samples identified 609 uremic solutes. None of the 609 uremic solutes were significantly different in concentration between the Itch patients and the No Itch patients in either the plasma or ultrafiltrate using the false discovery rate. PCA also failed to reveal a combination of solutes that was different between the Itch and No Itch patients in the plasma or ultrafiltrate, as illustrated in Fig 2. Including all 1,548 identified solutes also revealed no significant difference between the Itch and No Itch patients (S3 Fig).

## Discussion

Many patients with ESRD experience pruritus, a symptom which greatly impairs quality of life and is associated with increased mortality [1, 3]. Treatment has until recently been largely ineffective and the cause of pruritus is not known. The evidence for commonly implicated clinical lab parameters, such as phosphorus and parathyroid hormone, have so far been conflicting [3]. While its mechanism is likely complex, pruritus may be in part due to the waste solutes that accumulate in the plasma when the kidneys fail. That such uremic solutes contribute to pruritus is supported by the resolution observed after kidney transplantation [4]. Previous studies have suggested moreover that patients with shorter dialysis vintage are less likely to have pruritus [1]. Such patients are more likely to have residual kidney function that would keep plasma levels of the presumed causative solutes low [12]. No relation of pruritus with vintage was observed in the current study, but we excluded patients with significant residual function.

The current study used the largest metabolomic platform available to compare the plasma of hemodialysis patients with severe itch versus those with mild/no itch. Despite the use of a large metabolomic platform and the wide separation of pruritus severity in the two groups of patients, no solutes associated with pruritus were identified. This however does not exclude the possibility that uremic solutes contribute to pruritus. Such solutes may have escaped detection in our study for several reasons. First and perhaps most important, no analysis covers the whole range of solutes present in the plasma. In particular “metabolomics” analyses such as the one employed in the current study identify only small solutes with mass largely in the range < 1,000 Dalton [13]. Our study would thus fail to detect low molecular weight proteins or peptides which promote pruritus. Even among small solutes, metabolomic analysis can identify only those that are included in the chemical library of the metabolomic platform employed [14]. Small solutes that cause pruritus may not have been included in the platform that we used. A particular group of solutes which had limited representation in the platform we employed includes fatty acids which are modified in a variety of ways in kidney failure [15]. A further possibility is that pruritus may not be caused by a specific solute but rather by a combination of solutes with related chemical structure or biological function/origin [16]. Principal component analysis did not reveal such a combination in our study. A more sophisticated analysis of plasma from a larger number of patients might however identify related chemical structures which can trigger pruritus. In this regard, a previous metabolomic analysis did show separation in the solute profiles of patients with more and less pruritus but did not identify individual solutes or common chemical structures with which pruritus was associated [17]. Finally, the measurement of pruritus is subjective. Reported pruritus scores may be influenced by other co-morbidities and symptoms of patients. Of note, the Itch patients reported a greater overall symptom burden than the No Itch patients in our study. Environmental factors such as geographic region and seasonal changes may also affect pruritus severity [3]. Our patient population was from only Northern California, which may have limited our ability to identify culprit solutes.

Interest in the possibility that retained solutes could cause uremic pruritus has been stimulated by the discovery of itch sensory neurons [18, 19]. Signals originating in afferent fibers of these neurons in the skin are transmitted through their cell bodies in dorsal root ganglia to interneurons in the dorsal horn of the spinal cord. Complex processing in the spinal cord results in transmission of signals to the brain which provoke the sensation of itching and the response of scratching. The itch sensory neurons are similar in structure to nociceptive neurons which sense painful stimuli and heat and cold [18, 19]. Afferent terminals and dorsal root ganglia of itch sensory neurons however possess specific receptors for chemical stimuli which trigger itch. Particularly notable among these are Mas-related G protein-coupled receptors (Mrgprs) that respond to a variety of itch-inducing chemicals including the drug chloroquine and naturally occurring bile acids [20]. Occupation of these receptors opens ion channels which may also be directly activated by other small molecules including capsaicin. Of note, larger molecules including several cysteine proteases also bind to Mrgprs. This suggests as noted above that the search for solutes which cause uremic pruritus should not be confined to small molecules. The possibility that pruritus may be treated by agents which block receptors on primary itch neurons is being actively investigated. Recent promising treatments for uremic pruritus however target opioid receptors involved in the interneuronal processing of pruritus signals in the spinal cord [21, 22].

Pruritus in liver failure provides an interesting comparison to pruritus in kidney failure. Plasma accumulation of waste solutes has been considered responsible in both conditions [23]. Bile acids have been most frequently proposed as mediators of pruritus in liver disease, and a recent study showed that bile acids can activate the Mrgprs of itch sensory neurons [24]. Although bile acids are excreted mostly by the liver and intestines, they are partly cleared by the kidneys through glomerular filtration and tubular secretion [25]. As a result, plasma levels of bile acids are increased in kidney failure although to a much lesser degree than in patients with liver failure [26, 27]. Studies of the relation of total bile acid levels to pruritus in kidney failure patients have yielded inconsistent results [26, 28]. The metabolomic platform employed in our study included a limited number of bile acids, but we did not find any association of their levels with pruritus.

In conclusion, a metabolomic analysis of hemodialysis patients did not identify any solutes associated with pruritus. A limitation is that solutes causing pruritus may not be included in the chemical library of the metabolomic platform employed. A role for uremic solutes in pruritus remains to be established.

## Supporting information

S1 Fig Histogram of VAS scores. (PDF)

S2 Fig Relation of age with serum creatinine level and nPCR. (PDF)

S3 Fig Principal component analysis of all solutes in plasma and ultrafiltrate of hemodialysis patients. (PDF)

S1 Table Seasonal timing of study participation. (PDF)

S1 Dataset Metabolomics dataset in plasma and ultrafiltrate of hemodialysis. (XLSX)
