# Supplementary material for: Metabolomic analysis of uremic pruritus in patients on hemodialysis
Source: PLoS One. 2021 Feb 12;16(2):e0246765. doi: 10.1371/journal.pone.0246765 (PMC7880487; doi:10.1371/journal.pone.0246765)
Supplement: S1 Fig — (PDF) [file pone.0246765.s001.pdf]

S1 Fig – Histogram of VAS Scores

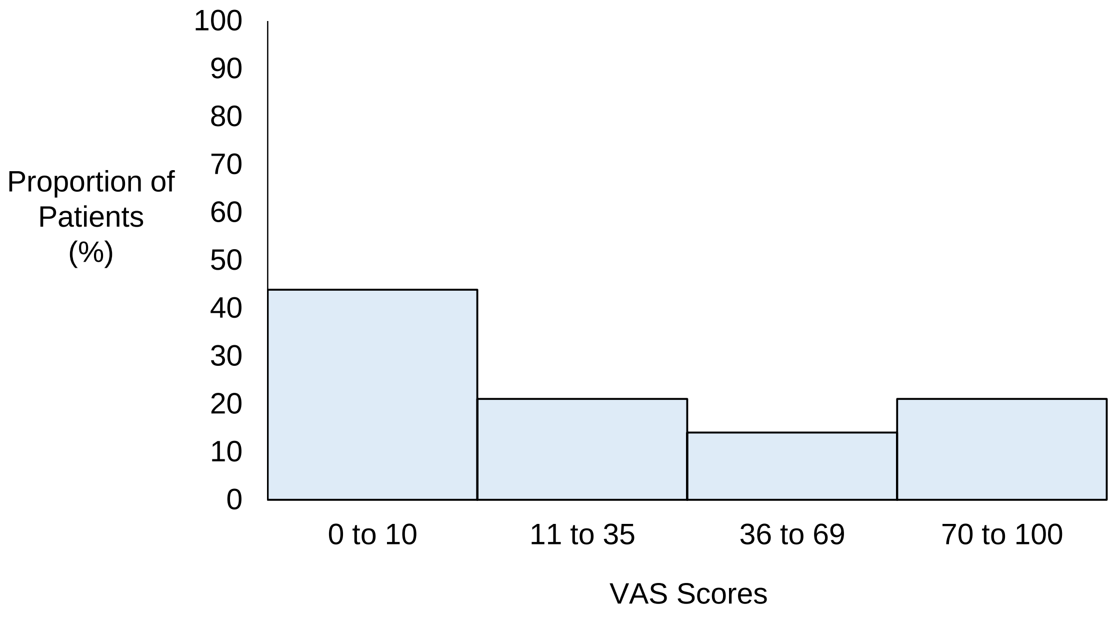

Histogram of the VAS scores for the 57 hemodialysis patients is illustrated.
