# Supplementary material for: Metabolomic analysis of uremic pruritus in patients on hemodialysis
Source: PLoS One. 2021 Feb 12;16(2):e0246765. doi: 10.1371/journal.pone.0246765 (PMC7880487; doi:10.1371/journal.pone.0246765)
Supplement: S2 Fig — (PDF) [file pone.0246765.s002.pdf]

S2 Fig – Relation of Age with Serum Creatinine Level and nPCR

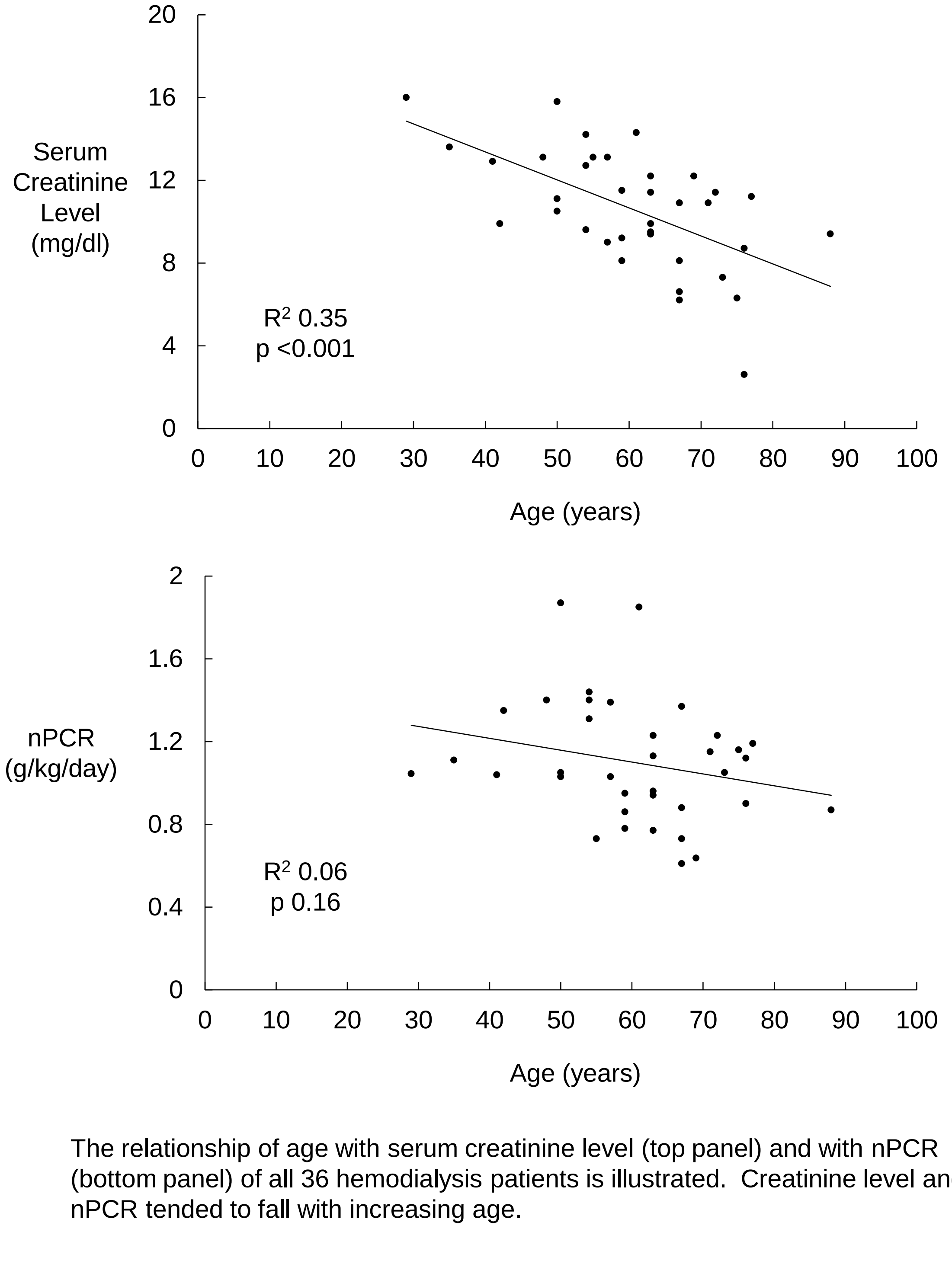

The relationship of age with serum creatinine level (top panel) and with nPCR (bottom panel) of all 36 hemodialysis patients is illustrated. Creatinine level and nPCR tended to fall with increasing age.
