# Supplementary material for: Metabolomic analysis of uremic pruritus in patients on hemodialysis
Source: PLoS One. 2021 Feb 12;16(2):e0246765. doi: 10.1371/journal.pone.0246765 (PMC7880487; doi:10.1371/journal.pone.0246765)
Supplement: S3 Fig — (PDF) [file pone.0246765.s003.pdf]

S3 Fig – Principal Component Analysis of All Solutes in Plasma and Ultrafiltrate of Hemodialysis Patients

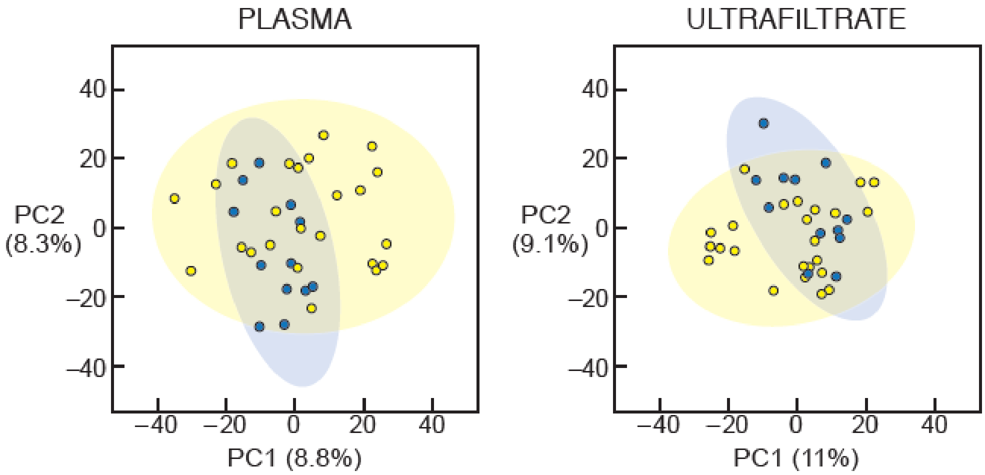

Principal component analysis score plots of the metabolomic solute profile in the plasma (left panel) and ultrafiltrate (right panel) are illustrated. There was no difference in the metabolomic profile of all 1,548 solutes between the 12 Itch patients (blue circles) and the 24 No Itch patients (yellow circles). PC1, principal component 1; PC2, principal component 2.
