# Supplementary material for: Metabolomic analysis of uremic pruritus in patients on hemodialysis
Source: PLoS One. 2021 Feb 12;16(2):e0246765. doi: 10.1371/journal.pone.0246765 (PMC7880487; doi:10.1371/journal.pone.0246765)
Supplement: S1 Table — (PDF) [file pone.0246765.s004.pdf]

S1 Table – Seasonal Timing of Study Participation

	Itch (n=12)	No Itch (n=24)	p value
Spring (#, %)	2 (17)	4 (17)	1.0
Summer (#, %)	2 (17)	5 (21)	0.77
Autumn (#, %)	1 (8)	6 (25)	0.23
Winter (#, %)	7 (58)	9 (38)	0.24

Seasonal timelines were based on the Northern Hemisphere. Characteristics between the two groups were compared using the chi-squared test.
